# Fibroblast GATA-4 and GATA-6 promote myocardial adaptation to pressure overload by enhancing cardiac angiogenesis

**DOI:** 10.1007/s00395-021-00862-y

**Published:** 2021-04-19

**Authors:** Gesine M. Dittrich, Natali Froese, Xue Wang, Hannah Kroeger, Honghui Wang, Malgorzata Szaroszyk, Mona Malek-Mohammadi, Julio Cordero, Merve Keles, Mortimer Korf-Klingebiel, Kai C. Wollert, Robert Geffers, Manuel Mayr, Simon J. Conway, Gergana Dobreva, Johann Bauersachs, Joerg Heineke

**Affiliations:** 1 Department of Cardiology and Angiology, Hannover Medical School, 30625 Hannover, Germany; 2 Department of Cardiovascular Physiology, European Center for Angioscience (ECAS), Medical Faculty Mannheim of Heidelberg University, 68167 Mannheim, Germany; 3 Shanghai Tianyou Hospital Affiliated To Tongji University, Shanghai, 200333 China; 4 Department of Anatomy and Developmental Biology, European Center for Angioscience (ECAS), Medical Faculty Mannheim of Heidelberg University, 68167 Mannheim, Germany; 5 Genome Analytics, Helmholtz Center for Infection Research, 38124 Braunschweig, Germany; 6 King’s British Heart Foundation Centre, King’s College London, London, UK; 7 HB Wells Center for Pediatric Research, Indiana University School of Medicine, Indianapolis, IN 46202 USA; 8 German Center for Cardiovascular Research (DZHK), Partner site Heidelberg/Mannheim, Germany; 9 Cardiovascular Physiology, European Center for Angioscience (ECAS), Medizinische Fakultät Mannheim, Universität Heidelberg, Ludolf-Krehl-Str. 7-11, 68167 Mannheim, Germany

**Keywords:** Cardiac remodeling, Fibroblast, Angiogenesis, Intercellular crosstalk

## Abstract

Heart failure due to high blood pressure or ischemic injury remains a major problem for millions of patients worldwide. Despite enormous advances in deciphering the molecular mechanisms underlying heart failure progression, the cell-type specific adaptations and especially intercellular signaling remain poorly understood. Cardiac fibroblasts express high levels of cardiogenic transcription factors such as GATA-4 and GATA-6, but their role in fibroblasts during stress is not known. Here, we show that fibroblast GATA-4 and GATA-6 promote adaptive remodeling in pressure overload induced cardiac hypertrophy. Using a mouse model with specific single or double deletion of *Gata4* and *Gata6* in stress activated fibroblasts, we found a reduced myocardial capillarization in mice with *Gata4/6* double deletion following pressure overload, while single deletion of *Gata4* or *Gata6* had no effect. Importantly, we confirmed the reduced angiogenic response using an in vitro co-culture system with *Gata4/6* deleted cardiac fibroblasts and endothelial cells. A comprehensive RNA-sequencing analysis revealed an upregulation of anti-angiogenic genes upon *Gata4/6* deletion in fibroblasts, and siRNA mediated downregulation of these genes restored endothelial cell growth. In conclusion, we identified a novel role for the cardiogenic transcription factors GATA-4 and GATA-6 in heart fibroblasts, where both proteins act in concert to promote myocardial capillarization and heart function by directing intercellular crosstalk.

**Supplementary Information:**

The online version contains supplementary material available at 10.1007/s00395-021-00862-y.

## Introduction

With continuous increase of life expectancy, preventing the progression of cardiovascular diseases has become the major challenge to preserve a high quality of life in the prolonged life span. Despite enormous efforts to improve preventive and therapeutic medical interventions, cardiovascular diseases remain the main cause of hospital admissions and death in developed countries [42]. Current treatment approaches for heart failure target predominantly cardiac myocytes as main responsible cell type for disease progression. Indeed, in response to pathological overload or ischemia, cardiomyocytes exert profound changes in gene expression, cellular hypertrophy and alterations in excitation contraction coupling that aggravate heart failure [13]. However, a new paradigm emerged in recent years, whereby also other cardiac cell types, especially endothelial cells, inflammatory cells and fibroblasts are on one hand strongly influenced by pathological insults and on the other hand heavily affect the disease process by interacting among each other and with cardiomyocytes [41]. How this crosstalk between different cardiac cell types is regulated, which mediators are involved, and how it affects the heart’s response to overload remains in large parts elusive.

Cardiac fibroblasts are among the non-myocytes that are increasingly recognized for their versatile function in the heart during pathological stress. While they were long neglected as sole provider of extracellular matrix, recent studies revealed that fibroblasts promote cardiomyocyte proliferation during development, promote adaptation through the secretion of IGF-1, impact hypertrophy in adult mice during pressure overload and regulate cardiac immune cell recruitment as well as scar remodeling after myocardial infarction [15, 16, 32, 34, 39]. Interestingly, cardiac fibroblasts exert a unique transcriptome, because they express high levels of cardiogenic transcription factors, such as *Tbx20*, *Gata4*, *Gata6*, and *Hand2*, which are all completely absent in skin fibroblasts. This indicates a specific differentiation pattern of cardiac fibroblasts designated to their operational area in the heart [8]. The functional impact of the cardiogenic gene program in myocardial fibroblasts, for example during myocardial overload, however, remains unknown.

The GATA family of transcription factors is divided into two groups based on their expression patterns, where GATA-1/-2/-3 are mainly found in the hematopoietic lineage, while GATA-4/-5/-6 are expressed in mesodermal derived tissues [26, 27]. In the developing and adult heart, the transcription factors GATA-4 and GATA-6 are important regulators for the expression of cardiac genes. Combined global loss of GATA-4 and GATA-6 prevents cardiomyocyte differentiation in early embryonic development resulting in acardia and embryonic death [51]. Cardiomyocyte specific deletion of GATA-4 or GATA-6 in adult mice leads to heart failure during pathological pressure overload [28, 44]. Both factors exert in part redundant functions in cardiac myocytes during stress, as they both promote cardiac hypertrophy and preserve heart function during overload. On the other hand, they contribute unique regulatory aspects for cellular gene-expression: GATA-4, but not GATA-6, for instance, drives a paracrine gene-program in cardiomyocytes to induce angiogenesis in adjacent endothelial cells [12, 43].

In the current study, we focused on the role of GATA-4 and GATA-6 as part of the cardiogenic gene-program in cardiac fibroblasts. We generated a mouse model of single and compound deletion of both factors in activated fibroblasts. We demonstrate here that single deletion of GATA-4 or GATA-6 in fibroblasts had no effect, while their compound deletion reduced the myocardial angiogenic response and thereby impaired heart function during cardiac pressure overload. Comprehensive RNA-sequencing from isolated heart fibroblasts lacking either *Gata4*, *Gata6* or both, revealed upregulation of several anti-angiogenic factors like *Angpt4*, *Thbs1*, and *Cd36* upon *Gata4/6* double deletion. Importantly, the anti-angiogenic effects of *Gata4/6* depleted fibroblasts were blunted by siRNA mediated downregulation of the fibroblast derived angiogenic inhibitors. Hence, we propose that the cardiogenic gene-program in heart fibroblasts maintains the high capillary density in the myocardium during pressure overload by directing intercellular communication towards endothelial cells.

## Materials and methods

### Animal experiments

To obtain mice with fibroblast-specific *Gata4*, *Gata6* or *Gata4/6* deletion, previously described *Gata4*^*flox/flox*^ mice (*Gata4*^*tm1.1Sad*^) [48] and *Gata6*^*flox/flox*^ mice (*Gata6*^*tm2.1Sad*^) [37] were interbred to obtain *Gata4/6*^*flox/flox*^ mice, as well as with mice expressing the Cre recombinase linked to a partial *Postn* promoter, *Tg*(*Postn-Cre*)*1Sjc* [23]. Littermate *Gata4*^*flox/flox*^, Gata6^flox/flox^ and *Gata4/6*^*flox/flox*^ mice were used as controls.

Pressure overload was induced in 8–10-week-old mice by constriction of the transverse aorta following standard procedures [11, 49]. All animal procedures were approved by the local state authorities (33.14-42502-04-13/1159).

Transverse aortic constriction (TAC) was induced in 8–10 week-old mice following standard procedure and maintained for 2 weeks (short TAC) or 6 weeks (long TAC) as indicated in the figures. Anesthesia was induced with isoflurane in an induction chamber with 3% and maintained at 2% via mask ventilation. Analgesia was provided by subcutaneous injection. After secured tracheal intubation, the aortic arch was visualized through partial upper thoracotomy and a 7–0 silk ligature was tied around a 27-gauge needle between the right brachiocephalic and left common carotid arteries. In sham treated animals, the ligation was not tied. During surgery, the animals were placed on a heating pad connected to a temperature controller to maintain body temperature. Postoperative analgesia was additionally provided in the drinking water.

### Echocardiography

Mice were anesthetized with 4% isoflurane in an induction chamber and placed on a heating pad to keep body temperature at 37 °C. Anesthesia was maintained with 1–2% isoflurane through a mask. Echocardiography was performed in short and long axis views using the Vevo 770 Visualsonics system. Quantification of cardiac function was performed using the cardiac package provided with the Vevo software.

### Histology

For organ harvesting, mice were sacrificed and whole hearts were immediately removed from the chest cavity, relaxed in 0.5 M KCl, washed in cold PBS, transversely dissected at the midventricular level and immediately embedded in Tissue-Tek OCT compound (Sakura). Slices of 12 µm thickness were stained with Picro Sirius red following the standard protocol. Fibrosis was quantified as the fraction (in %) of fibrotic tissue (stained in red) per total area of the myocardium using Adobe Photoshop imaging software. Perivascular fibrosis ratio (PFR) was determined as perivascular fibrotic area per vessel area using Adobe Photoshop imaging software.

### Immunofluorescence staining

Immunofluorescence staining was performed on 7 µm OCT cryosections. Slides were fixed with 4% PFA for 20 min, permeabilized in 0.3% Triton-X for 20 min and blocked in 3% BSA for at least 1 h at room temperature. Each step was followed by three washing steps in PBS. Slides were then serially incubated with primary and secondary antibodies and mounted with Vectashield Mounting Medium with DAPI. The following primary antibodies were used: anti-GATA-4 (1:50, Santa Cruz, sc-1237), anti-GATA-6 (1:20, R and D Systems, AF1700), anti-PDGFR-α (1:50, R and D Systems, AF1062), and anti-alpha smooth muscle Actin (1:400, Abcam, ab7817). Secondary antibodies (Alexa Fluor 488 and 555) anti-goat were used in a 1:200 dilution, anti-mouse (Alexa Fluor 555) in a 1:800 dilution.

Staining of the cell membrane and capillaries was performed with wheat germ agglutinin Texas red (Invitrogen, w21405) and fluorescein-labelled Isolectin B4 (IB4, Vector laboratories, FL-1201), respectively, according to the manufacturer’s protocols. The ratio of IB4-stained capillaries per cardiomyocyte was assessed in three high power fields (200 × magnification) per heart to determine capillary density. Images were acquired with an Axiovert microscope (Carl Zeiss, Jena, Germany). Whole heart brightfield and fluorescence images were acquired on a Zeiss Axio Scan System.

### Cell culture

Human umbilical vein endothelial cells (HUVEC) and human cardiac microvascular endothelial cells (HCMEC) were purchased from PromoCell and cultured in Endothelial Cell Growth Medium (PromoCell) supplemented with growth factor cocktail containing FCS 2%, ECGS 0.4%, EGF 0.1 ng/ml, bFGF 1 ng/ml, Heparin 90 µg/ml, and Hydrocortisone 1 µg/ml. Neonatal rat ventricular fibroblasts were isolated from 1 to 3 day-old Sprague–Dawley rats and separated from cardiomyocytes by Percoll density gradient centrifugation as previously described [7, 50]. Isolated fibroblasts were cultured in DMEM containing 10% FCS and antibiotics, until they were switched to media with 2% FCS or no FCS for experiments.

Transfection of rat cardiac fibroblasts was performed at 80% confluency after a maximum of two passages. 100 nM of siRNA (siGATA4, J-090725-09-0010, Dharmacon; siGATA6, J-080135-13-0010, Dharmacon; siCD36, SASI_Rn02_00264778, Sigma–Aldrich; siAngp4, J-093010-09-0002, Dharmacon; neg. Ctrl, AM4635, Ambion) were incubated for 20 min with 32 µl Lipofectamine 2000 in Opti-MEM before the mixture was added to the cells for 5 h. Subsequently the medium was changed to DMEM 2% FCS and cells were cultured for 48 h before conducting experiments.

Scratch assays were performed in 24-well plates after HUVECs or HCMECs reached > 90% confluency. The cell monolayer was scraped in a straight line with a p200-pipet tip to create a “scratch injury”. The debris was removed by washing the cells once with PBS and fresh medium was added together with transfected fibroblasts cultured in permeable inserts or recombinant protein (thrombospondin-1 human, 50 nM, ECM002 Sigma; human angiopoietin-4, 50 ng/ml, 964-AN-025 R and D Systems) as indicated in the figure. Pictures were taken immediately after scratch, as well as 6 h and 10 h later. The scratch area was analyzed using ImageJ software and migration was calculated as the difference in cell-free area after 6 or 10 h compared to 0 h relative to the initial scratch area.

Tube formation assay was performed in matrigel coated 96-well plates. HUVECs were either co-cultured with transfected fibroblasts or treated with recombinant thrombospondin-1 or angiopoietin 4 (concentration as indicated above). Pictures were taken after 8 and 24 h with 50-fold magnification and closed circular structures were quantified as tubes using ImageJ software.

### Isolation of murine cardiac cells

For culture, cardiac fibroblasts were isolated from 6 to 8 week-old *Gata4*^*flox/flox*^, *Gata6*^*flox/flox*^, and *Gata4*^*flox/flox*^*Gata6*^*flox/flox*^ mice as previously described [34]. Whole hearts without atria were quickly cut into small pieces in ice-cold PBS. Digestion was performed using Liberase TH (Roche) with SADO buffer mix solution (20 mM HEPES–NaOH (Roth, Nr. 9105, pH 7.6), 130 mM NaCl (Roth, Nr. 9265), 3 mM KCl (Roth, Nr. 6781), 1 mM NaH2PO4 (Sigma, Nr. S5011), 4 mM Glucose (Roth, Nr. HN06), 3 mM MgSO4 (Roth, Nr. 2278) in sterile, filtered dH20). The digested cell solution was plated and after 3 h washed once with PBS and changed to DMEM supplemented with 10% FCS. Isolated fibroblasts were infected with AdCre or Adβ-Gal for 3 h and after two washing steps with PBS cultured in DMEM supplemented with 2% FCS for 48 h. Cells were passaged two times at maximum before harvesting.

For immediate RNA or protein-isolation, cardiac fibroblasts and cardiac endothelial cells were isolated from hearts of adult mice using CD146 microbeads and feeder removal microbeads with MACS (magnetic cell separation) technology from Miltenyi Biotec.

Adult ventricular cardiomyocytes were isolated as previously described using a Langendorff system [24].

### RNA isolation and deep-sequencing analysis

RNA from murine cardiac fibroblasts was isolated using the NucleoSpin RNA kit (Macherey–Nagel) according to the manufacturer’s protocol. Further analysis was performed at the Helmholtz Center for Infection Research in Braunschweig. Quality and integrity of total RNA were controlled on the Agilent Technologies 2100 Bioanalyzer. The RNA-Seq library was generated from 500 to 1000 ng total RNA using Dynabeads mRNA DIRECT Micro Purification Kit (Thermo Fisher Scientific) for mRNA purification followed by ScriptSeq v2 RNA-Seq Library Preparation Kit (Epicentre) according to the manufacturers’ protocols. The libraries were sequenced on Illumina HiSeq2500 using TruSeq SBS Kit v3-HS (50 cycles, single-ended run) with an average of 3 × 10^7^ reads per RNA sample. Before alignment to the reference (mm10), each sequence was trimmed on base call quality and sequencing adapter contamination using the Trim Galore! wrapper tool (http://github.com/felixkrueger/trimgalore). Reads shorter than 20 nt were removed from FASTQ files. Trimmed reads were aligned to the reference using the short-read aligner STAR (https://code.google.com/p/rna-star/). Feature counts were determined using R package “Rsubread”. Only genes showing counts greater 5 at least two times across all samples were considered for further analysis (data cleansing). Gene annotation was done by R package “bioMaRt”. Library size normalized counts (cpm counts per million) were log2 transformed and further normalized according to 50th percentile (quartile normalization using edgeR). Differential gene expression was calculated by R package “edgeR”. Differentially expressed genes were selected with 0.5 < fold change > 2 and FDR < 0.05. GO biological process clusters from different groups of genes were performed by Metascape [52]. Cluster relations were visualized in a network plot using cytoscape [35]. Heatmaps of differentially regulated genes were generated by using heatmap.2 function in ggplot2 library in R. Our data set of RNA-Seq data is deposited in the National Center for Biotechnology Information’s Gene Expression Omnibus database under accession number GSE155358.

### Quantitative real-time PCR

Total RNA from fibroblasts of adult mouse hearts was isolated using the NucleoSpin RNA II Kit (Macherey Nagel) following the manufacturer’s protocol. cDNA was generated using the Maxima H Minus First Strand cDNA Synthesis Kit (Thermo Fisher Scientific) and quantitative PCR was performed following standard procedures. Gene-expression was normalized to *Rpl7* or *Gapdh* mRNA expression as indicated in the figure legend. Absolute quantification of *Gata* factor mRNA was based on standard curves with plasmids containing the full-length *Gata* sequences. Primer sequences are provided in Supplemental Table 1.

### Western blot

Protein was extracted from fibroblasts of adult mouse hearts or whole heart tissue of *Gata4/6-Per-Cre* or *Gata4/6* control mice as indicated, separated on an SDS–polyacrylamide gel under reduced denaturing conditions and subsequently blotted onto a PVDF membrane following standard procedures. Applied primary antibodies were anti-GATA-4 (1:1000, Santa Cruz, sc-1237), anti-GATA-4 (1:100, Santa Cruz, sc-25310), anti-GATA-6 (1:1000, R and D systems, AF1700) anti-GAPDH (1:3000, Fitzgerald, 10R-G109a) or anti-Actin (1:10,000, Sigma-Aldrich, A2066). Protein levels were quantified using Quantity One software (Bio-Rad).

### Extracellular matrix isolation and proteomic analysis

Enrichment of extracellular matrix proteins was performed as previously described [1, 2]. Heart tissue samples of *Gata4/6-Per-Cre* or *Gata4/6* control mice were sequentially incubated with 0.5 M NaCl (1 h), 0.1% SDS (16 h) and 4 M guanidine hydrochloride (48 hours). After precipitation of GuHCl extracts, protein samples were enzymatically deglycosylated and subjected to in-solution trypsin digestion. Digested peptides from GuHCl extracts were injected for liquid chromatography-tandem mass spectrometry (LC-MS/MS) analysis onto a Q Exactive HF mass spectrometer (Thermo Fisher Scientific) for discovery proteomics. PC analysis was achieved using factoextra package, and visualized with ggplot2 library in R. A clustered heatmap that indicates significantly different protein levels of main ECM components was created by pheatmap library in R.

### Statistical analysis

Data are presented as mean ± SD. The investigators were blinded for mouse genotype during surgery, echocardiography, organ weight determination, and all histological and immunofluorescence quantifications. For comparison of two groups, unpaired two-tailed Student’s *t* test was used to determine statistical significance. For comparison of more than two groups, statistical significance was determined using one-way ANOVA (with Dunnett’s multiple-comparisons test or Tukey’s multiple-comparisons test) for one independent variable and two-way ANOVA (with Sidak’s multiple-comparisons test) for two independent variables with GraphPad Prism 7 software. *P* values less than 0.05 were considered statistically significant.

## Results

### Among the GATA transcription factors, *Gata4* and *Gata6* show the highest expression in cardiac fibroblasts

**Fig. 1:**
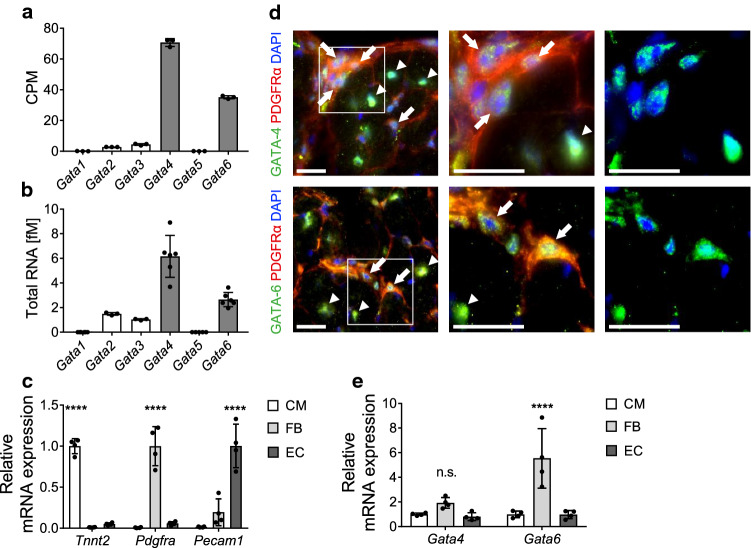
*Gata4* and *Gata6* show the highest expression among GATA family members in cardiac fibroblasts. **a**, **b** Quantification of *Gata*-factor RNA expression levels in isolated cardiac fibroblasts from RNA-sequencing [shown as counts per million reads (CPM, **a**)] and quantitative real-time-PCR (**b**) demonstrates that *Gata4* and *Gata6* are the highest expressed GATA family transcription factors in cardiac fibroblasts. RNA expression levels are normalized to *Rpl7* (**b**). **c** Quantitative real-time-PCR of cell-type specific marker genes shows high purity of isolated cardiomyocytes (CM), fibroblasts (FB) and endothelial cells (EC) from adult mouse hearts. **d** Representative immunofluorescence images from transverse mouse heart sections show GATA-4 (upper images, green) and GATA-6 (lower images, green) in cardiac fibroblasts (stained with PDGFRα, red), nuclei are stained with DAPI (blue). Arrows indicate GATA-4 or GATA-6 positive cardiac fibroblasts, arrowheads indicate GATA-4 or GATA-6 positive cardiomyocytes. Scale bar 20 µm. **e** Quantitative real-time-PCR shows RNA expression level of *Gata4* and *Gata6* in FB compared to CM and EC. RNA expression levels are normalized to *Gapdh* (**c**, **e**). Data are shown as mean ± SD. Two-way ANOVA with Sidak’s multiple-comparisons test was used to test for significant differences. n.s. indicates no statistical significance between groups, ****p < 0.0001

First, we analyzed the mRNA expression levels of all GATA family transcription factors in cardiac fibroblasts isolated from adult mice. The results from bulk RNA-sequencing of wild-type cardiac fibroblasts showed 16-fold higher counts of *Gata4* and eightfold higher counts of *Gata6* per million reads compared to *Gata3*, which showed the third highest expression level (Fig. 1a). In a second approach, we analyzed the absolute expression level of each GATA factor by quantitative real-time PCR (qPCR) from isolated heart fibroblasts. We found a similar expression pattern as revealed by RNA sequencing, with the highest expression level for *Gata4* (around 6 fM) and *Gata6* (around 3 fM), significantly lower RNA amounts of *Gata2* and *Gata3* (below 2 fM), and negligible amounts of *Gata1* and *Gata5* (Fig. 1b). Evaluation of cell purity showed high enrichment of cell-type specific marker genes in fractions of isolated adult cardiomyocytes (CM), fibroblasts (FB), and endothelial cells (EC), indicating that the described *Gata* factor expression level are indeed resulting from cardiac fibroblasts (Fig. 1c). Immunofluorescence co-staining for GATA-4 or GATA-6 and platelet-derived growth factor receptor alpha (PDGFRα), which serves as a marker for fibroblasts in cardiac tissue, confirms the localization of GATA-4 and GATA-6 in the nuclei of cardiac fibroblasts (Fig. 1d) in transverse mouse heart sections. Moreover, GATA-4 and GATA-6 staining also showed a strong signal in the nuclei of large PDGFRα-negative cells, which are most likely cardiomyocytes. In addition, comparison of the *Gata4* and *Gata6* mRNA expression levels in isolated cell fractions indicated fibroblasts to express at least equal *Gata4* levels, and even significantly higher *Gata6* levels compared to cardiomyocytes and endothelial cells (Fig. 1e).

### Deletion of *Gata4* and *Gata6* in activated cardiac fibroblasts aggravates heart failure after pressure overload

**Fig. 2:**
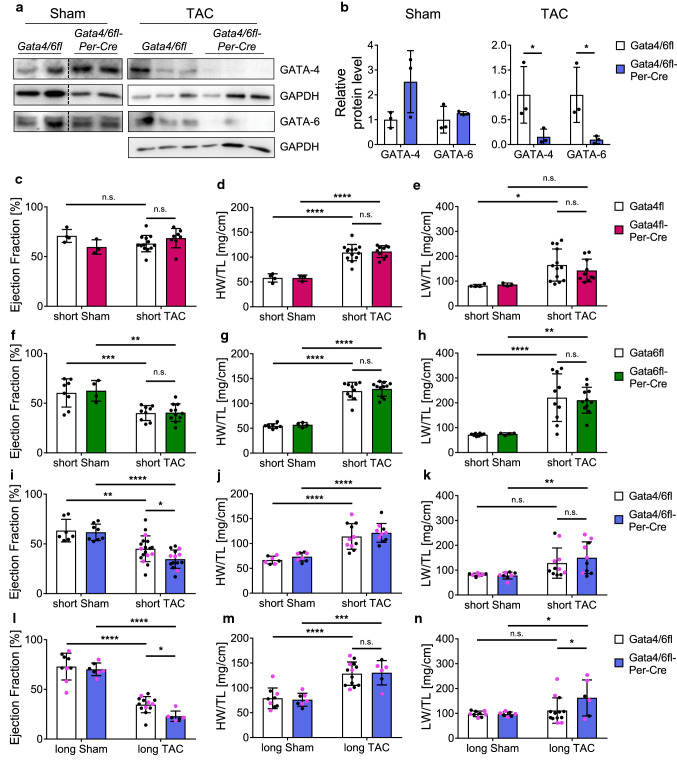
Combined deletion of *Gata4* and *Gata6* in activated cardiac fibroblasts aggravates heart failure after short- and long-term pressure overload. **a**, **b** Western blot of isolated cardiac fibroblasts from *Gata4/6fl-Per-Cre* and *Gata4/6fl* littermate control mice after pressure overload or sham (**a**) and quantification of relative protein levels normalized to GAPDH (**b**) showing efficient reduction of GATA-4 and GATA-6 protein levels after TAC in *Gata4/6fl-Per-Cre* mice. **c–e** Quantification of the ejection fraction (**c**), heart weight/tibia length ratio (HW/TL, **d)** and lung weight/tibia length ratio (LW/TL, **e**) in *Gata4fl-Per-Cre* and *Gata4fl* littermate control mice shows signs of compensated cardiac hypertrophy without significant differences in both groups after short-term pressure overload. **f–h** Quantification of the ejection fraction (**f)**, HW/TL (**g**), and LW/TL ratio (**h**) in *Gata6fl-Per-Cre* and *Gata6fl* littermate control mice shows decreased cardiac function without significant differences in both groups after short-term pressure overload. **i–n** Quantification of the ejection fraction (**i**, **l**), HW/TL (**j**, **m**), and LW/TL ratio (**k**, **n**) in *Gata4/6fl-Per-Cre* mice and *Gata4/6fl* littermate controls shows that mice with deletion of *Gata4* and *Gata6* in cardiac fibroblasts display a significantly worse cardiac function after short- and long-term pressure overload and an increased LW/TL ratio after long-term pressure overload compared to littermate control mice, while the HW/TL ratio is not significantly different. Selected animals for further histological analysis (see Fig. 3) are indicated in pink. Data are shown as mean ± SD. Two-way ANOVA with Sidak’s multiple-comparisons test was used to test for statistical significance. n.s. indicates no statistical significance between groups. *p < 0.05, **p < 0.01, ***p < 0.001, ****p < 0.0001

To investigate the functional relevance of *Gata4* and *Gata6* in activated fibroblasts in vivo, we interbred *Gata4*^*flox/flox*^ and *Gata6*^*flox/flox*^ mice with mice expressing the Cre recombinase linked to a partial periostin (*Postn*) promoter to obtain single deletion of *Gata4* (*Postn*^*Cre/*+^;* Gata4*^*flox/flox*^, short: *Gata4fl-Per-Cre*, Suppl Figure 1a pink) and *Gata6* (*Postn*^*Cre/*+^;* Gata6*^*flox/flox*^, short: *Gata6fl-Per-Cre*, Suppl. Figure 1a green), as well as combined deletion of *Gata4/6* (*Postn*^*Cre/*+^;* Gata4*^*flox/flox*^;* Gata6*^*flox/flox*^, short: *Gata4/6fl-Per-Cre*, Suppl. Figure 1a blue) in activated cardiac fibroblasts [23, 37, 48]. The *Per-Cre* mice were previously described as an efficient model to target gene expression in adult activated fibroblasts following cardiac injury, while quiescent fibroblasts or cardiomyocytes of the heart are predominantly unaffected [5, 16, 20, 21, 34, 39]. Immunoblot analysis of isolated fibroblasts from stressed hearts of *Gata4/6fl-Per-Cre* mice showed a marked reduction of over 80% for GATA-4 and over 90% for GATA-6 protein levels after pressure overload compared to littermate control mice (Fig. 2a, b), while total GATA-4 and GATA-6 levels in isolated fibroblasts of sham mice or whole heart tissue of these mice were not significantly affected (Fig. 2a, b and Suppl. Figure 1b). RNA levels of *Gata4* and *Gata6* in isolated fibroblasts from wild-type mice were unchanged after pressure overload compared to sham samples (Suppl. Figure 1c). Measurements of the heart weight/tibia length (HW/TL) and lung weight/tibia length (LW/TL) ratios of the *Gata4fl-Per-Cre* and *Gata6fl-Per-Cre* mice showed a marked increase in both groups after short-term (2 weeks) pressure overload by transverse aortic constriction (TAC), demonstrating a significant hypertrophic response of the heart with signs of pulmonary congestion (Fig. 2d, e, g, h). Assessment of cardiac function by transthoracic echocardiography showed no significant differences of the cardiac ejection fraction (EF) in *Gata4fl-Per-Cre* mice after short-term sham or TAC surgery compared to littermate control mice, indicating a sufficient compensation of the pressure overload in both groups (Fig. 2c, additional echocardiography parameters in Suppl. Table 2). The *Gata6fl-Per-Cre* mice showed a significantly decreased EF after short-term pressure overload compared to sham mice, but as the cardiac function of littermate control mice equally decreased after TAC, we found that single deletion of *Gata6* in cardiac fibroblasts is not sufficient to affect the cardiac function after pressure overload (Fig. 2f). Analysis of cardiac function, HW/TL and LW/TL ratios in *Wt-Per-Cre* mice did not reveal any significant influence of the *Per-Cre* in sham mice or after pressure overload (Suppl. Figure 1d–f). As previous studies demonstrated that GATA-4 and GATA-6 exert partially redundant functions in cardiomyocytes in case of single deletion of one of these factors, we next investigated the morphological and functional effects of a double deletion of *Gata4* and *Gata6* in activated cardiac fibroblasts [44, 51]. After short-term TAC, we found equally increased HW/TL and LW/TL ratios in *Gata4/6fl-Per-Cre* and littermate control mice compared to sham animals (Fig. 2j, k). However, the cardiac function of mice with *Gata4/6*-double deletion was significantly worse compared to littermate control mice after short-term pressure overload (Fig. 2i). This effect was maintained after prolonged exposure to pressure overload for 6 weeks (long-term) when cardiac function was further decreased in *Gata4/6fl-Per-Cre* and littermate control mice compared to the short time point, but remained significantly worse in *Gata4/6fl-Per-Cre* versus control animals (Fig. 2l). The HW/TL ratio was similarly increased in *Gata4/6fl-Per-Cre* mice and controls, while the LW/TL ratio was only increased in the *Gata4/6fl-Per-Cre* mice, but not in the littermate controls in this cohort, indicating aggravated pulmonary congestion due to cardiac dysfunction as consequence of reduced GATA-4 and GATA-6 levels in cardiac fibroblasts (Fig. 2m, n). Furthermore, qPCR analysis of a panel of fibrosis and cardiac embryonic genes revealed an increased mRNA expression of *Nppa* (ANP) in the myocardium of *Gata4/6fl-Per-Cre* versus control mice after long TAC, again as sign of aggravated heart failure in these mice (Suppl. Figure 1g).

Overall, we found that single deletion of *Gata4* or *Gata6* in activated cardiac fibroblasts did not significantly affect the cardiac response to pressure overload, while mice with double deletion of *Gata4/6* displayed a reduced cardiac function after short- and long-term, and more pulmonary congestion after long-term TAC compared to littermate controls. Therefore, our further analyses predominantly focus on *Gata4/6* double deletion rather than single deletion of *Gata4* or *Gata6*.

### Deletion of *Gata4/6* in cardiac fibroblasts reduces the capillary density in the heart after pressure overload

**Fig. 3:**
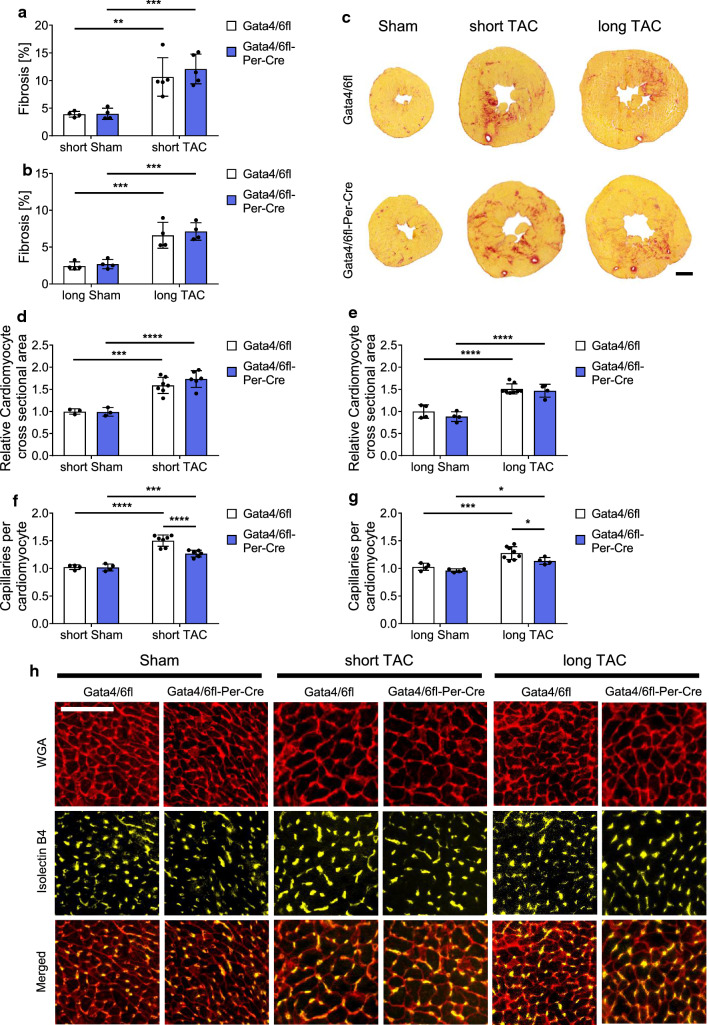
Mice with fibroblast-specific deletion of *Gata4* and *Gata6* show a reduced capillary density after pressure overload. **a–c** Quantification of cardiac fibrosis from transverse mouse heart sections stained with Sirius-red after short (**a**) and long (**b**) pressure overload and representative pictures of Sirius-red stained hearts (**c**), scale bar 1 mm. **d**, **e** Quantification of the cardiomyocyte cross-sectional area after short (**d**) and long (**e**) pressure overload. **f–h** Quantification of the capillary density displayed as capillary per cardiomyocyte ratio after short (**f**) and long (**g**) pressure overload and representative pictures of WGA and IsolectinB4 stained heart sections (**h**), scale bar 50 µm. Data are shown as mean ± SD. Two-way ANOVA with Sidak’s multiple-comparisons test was used to test for statistical significance. *p < 0.05, **p < 0.01, ***p < 0.001, ****p < 0.0001

To investigate myocardial structural changes upon fibroblast *Gata4/6* deletion, we used Sirius-red staining to detect fibrosis in response to short- or long-term pressure overload (mice that were selected for histological analysis are indicated in Fig. 2 i–n as pink datapoints). The fibrotic area significantly increased in *Gata4/6fl-Per-Cre* and control mice following short- and long-term TAC versus sham operated mice, but no differences were observed between both genotypes (Fig. 3a–c). Additional quantification of the perivascular fibrosis ratio (PFR) showed a similar increase of the PFR after short and long-term TAC compared to sham, while we did not observe any difference between *Gata4/6fl-Per-Cre* and control mice (Suppl. Figure 2a, b). We also quantified the number of fibroblasts per cardiomyocyte to detect potential differences in fibroblast cell numbers due to changes in developmental differentiation or proliferation. While the number of fibroblasts per cardiomyocyte significantly increased in response to short- and long-term pressure overload, we did not find significant changes between *Gata4/6fl-Per-Cre* and control mice (Suppl. Figure 2c). Furthermore, immunofluorescence staining for the leukocyte marker CD45 did not reveal any differences in the number of inflammatory cells between *Gata4/6fl-Per-Cre* and control mice (Suppl. Figure 2d). Moreover, quantification of α-smooth muscle actin positive vessels from immunofluorescence stainings did not indicate any impact of *Per-Cre* driven *Gata4/6* deletion on the amount of cardiac arterioles and arteries (Suppl. Figure 2e, f). Next, we determined the hypertrophic response and capillarization of cardiac tissue after staining for wheat germ agglutinin (WGA) and Isolectin B4 (IB4). As expected, the cardiomyocytes significantly increased in size in response to pressure overload as shown in Fig. 3d, e. Nevertheless, the cardiomyocyte cross-sectional area similarly increased in *Gata4/6fl-Per-Cre* and control mice, indicating that the cellular mechanisms enabling compensatory cardiac hypertrophy do not depend on the expression levels of *Gata4* and *Gata6* in cardiac fibroblasts. Quantification of the fibrotic area and the hypertrophic response in *Gata4* and *Gata6* single deleted mice and *Wt-Per-Cre* mice showed a similar increase after TAC compared to sham, while no differences occurred between the *Gata4* or *Gata6* single deletion or control mice (Suppl. Figure 3a–f). In contrast to the unaffected fibrotic and hypertrophic responses, we found that the increase in the number of capillaries per cardiomyocyte after short- and long-term TAC compared to sham was significantly reduced in *Gata4/6fl-Per-Cre* compared to control mice (Fig. 3f–h). Capillary density was unchanged in fibroblast *Gata4* single deleted mice or *Wt-Per-Cre* mice after TAC, or even increased in the *Gata6* single deleted mice (Suppl. Figure 3g–i). Sham operated *Gata4/6fl-Per-Cre* mice also showed no reduction in cardiac capillarization versus *Gata4/6fl* mice, because Cre activation and, therefore, *Gata4/6* deletion does only occur after TAC surgery in our model. Differences of the angiogenic response in control mice between the analyzed groups after TAC are most likely due to slight variation in the genetic mouse background, therefore, only littermate animals were used as respective controls for each group. The impaired angiogenic response upon combined *Gata4/6* deletion after TAC raised the question, how the deletion of the transcription factors *Gata4* and *Gata6* in cardiac fibroblasts could affect the formation of cardiac endothelial cells, which were not directly targeted in our in vivo model.

### In vitro co-culture of *Gata4/6*-deprived cardiac fibroblasts and endothelial cells shows reduced endothelial cell migration and tube formation

**Fig. 4:**
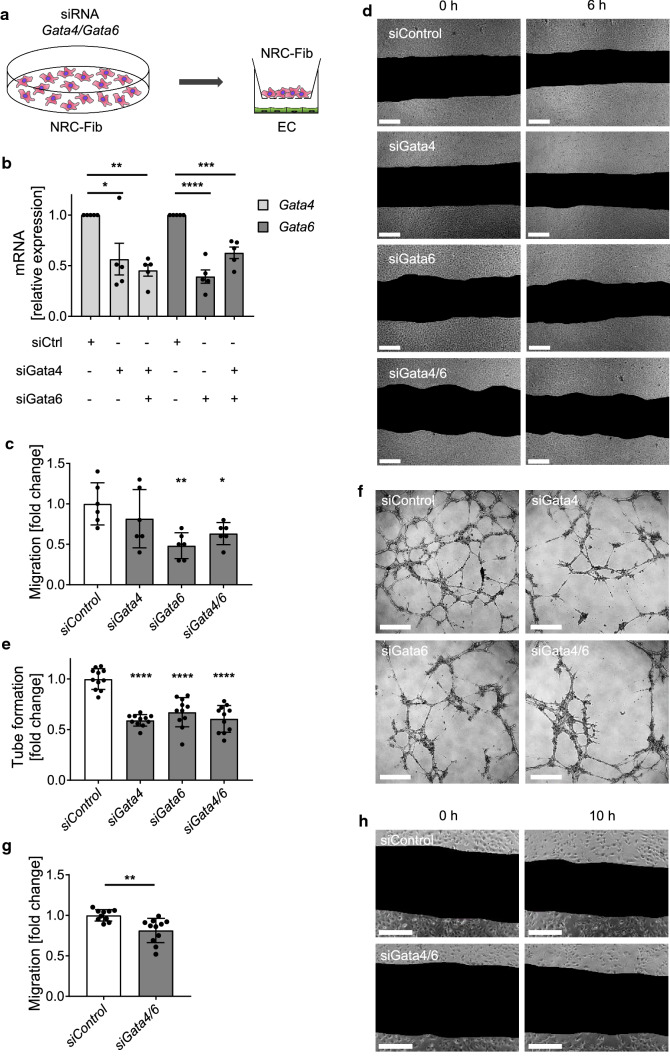
Endothelial cells exert reduced migration and tube formation in co-culture with fibroblasts treated with siRNA to downregulate *Gata4* and *Gata6* compared to control-treated fibroblasts. **a** Schematic overview of co-culture experimental setup. *Gata4* and *Gata6* were downregulated in neonatal rat cardiac fibroblasts (NRC-Fib) by siRNA transfection 48 h before co-culture with HUVEC or HCMEC. **b** Relative mRNA level of *Gata4* and *Gata6* in rat cardiac fibroblasts 48 h after siRNA transfection. RNA expression levels are normalized to *Gapdh.* **c**, **d** Quantification of endothelial cell (HUVEC) migration (**c**) and representative images (**d**) from scratch assays. Pictures were taken directly after scratch (0 h) and 6 h later. **e**, **f** Quantification of tube formation (**e**) and representative pictures (**f**) of HUVEC/Fibroblast co-cultures on a 3D matrigel matrix. Images for quantification of tube formation were taken after 8 h of co-culture. Quantification of cardiac microvascular endothelial cell (HCMEC) migration (**g**) and representative images (**h**). Pictures were taken directly after scratch (0 h) and 10 h later. Scale bar (**d**, **f**, **h**) 200 µm. Data are shown as mean ± SD. One-way ANOVA with Dunnett’s multiple-comparisons test (**b**, **c**, **e**) or unpaired *t* test (**g**) was used to test for statistical significance. *p < 0.05, **p < 0.01, ***p < 0.001, ****p < 0.0001

To address the finding that fibroblast-specific double-deletion of *Gata4* and *Gata6* reduces the angiogenic response in the heart after pressure overload, we established an in vitro co-culture system. Therefore, cardiac fibroblasts from newborn rats (1–3 days-old) were isolated and transfected with specific siRNAs to downregulate the expression of *Gata4*, *Gata6* or both. These targeted fibroblasts or control cells were cultured in permeable inserts on top of endothelial cells, allowing the exchange of soluble factors by diffusion in the culture medium (Fig. 4a). Due to the distance between the fibroblast covered inserts and the bottom of the cell culture dish below, direct cell–cell contact of fibroblasts and endothelial cells was prevented. Quantification of the mRNA expression level of *Gata4* and *Gata6* and protein level of GATA-4 and GATA-6 in cardiac fibroblasts after siRNA transfection demonstrated a downregulation of around 50% on RNA and protein level compared to fibroblasts transfected with siRNA control (Fig. 4b and Suppl. Figure 3j, k). The endothelial cells (HUVEC) showed a markedly reduced migration after 6 h of co-culture with *siGata6* or *siGata4/6* treated fibroblasts compared to controls, while co-culture with *siGata4* treated fibroblasts did not significantly affect the HUVEC migration (Fig. 4c, d). Another important parameter of endothelial activity is the ability to form a closed interaction network on matrigel matrix, which can be assessed in a tube formation assay. HUVECs that were co-cultured with *siGata4*, *siGata6* or *siGata4/6* transfected fibroblasts on matrigel built significantly less closed tube-structures compared to controls (Fig. 4e, f). To confirm the anti-angiogenic effect of *Gata4/6* deletion in fibroblasts on microvascular cells, we additionally conducted co-culture experiments with human cardiac microvascular endothelial cells (HCMEC) and *siGata4/6* or control treated fibroblasts. The HCMECs showed a significantly reduced migration after 10 h of co-culture with *siGata4/6* treated fibroblasts compared to controls (Fig. 4g, h). In conclusion, our in vitro experiments support the finding that fibroblast *Gata4/6* is required for an adaptive expansion of endothelial cells and that this intercellular crosstalk is presumably mediated by soluble factors secreted from cardiac fibroblasts.

### Simultaneous deletion of *Gata4/6* in cardiac fibroblasts changes the transcriptome towards higher expression of anti-angiogenic mediators

**Fig. 5:**
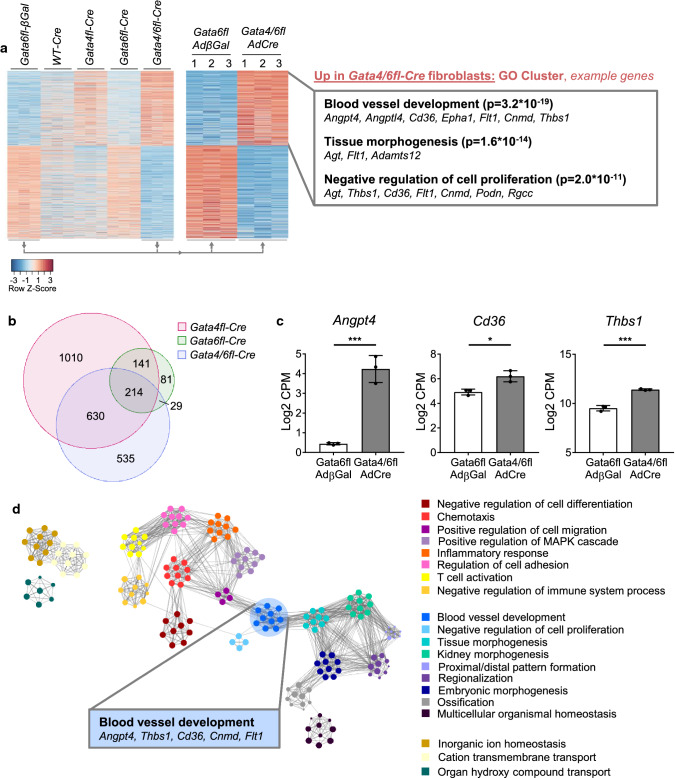
Transcriptome analysis of isolated cardiac fibroblasts showing an upregulation of anti-angiogenic genes in cells with *Gata4*/*Gata6* deletion compared to control cells. **a** Heatmap of differentially regulated genes showing all analyzed groups (left panel) and direct comparison of *Gata6fl-βGal* (control) vs. *Gata4/6fl-Cre* (right panel) isolated cardiac fibroblast samples (*n* = 3 in each group). Gene ontology (GO, biological process) classification and selected example genes are demonstrated on the right. Bioinformatic analysis of RNA-Seq data is described in the “Materials and methods” section. **b** Venn diagram of significantly upregulated genes in fibroblasts with *Gata4*, *Gata6*, and *Gata4/6* deletion compared to *Gata6fl-βGal* control samples. **c** RNA expression level of selected anti-angiogenic genes from RNA-sequencing. **d** Visualization of the top 20 GO BP clusters and relationships as a network plot. Each node represents an enriched term and is colored depending on its cluster ID, the cluster ID legend is shown on the right. Terms with a similarity > 0.3 are connected by edges. Data are shown as mean ± SD. Student’s *t* test was used for comparison of two groups. *p < 0.05, ***p < 0.001

To identify target genes of *Gata4/6* in cardiac fibroblasts that might influence the adaptive angiogenic response in the heart after pressure overload, we isolated fibroblasts from hearts of the previously described *Gata4fl*, *Gata6fl*, and *Gata4/6fl* as well as from wild-type mice. The isolated fibroblasts were cultured in vitro and subsequently the loxP targeted exons of the *Gata4* (exon 3–5) and the *Gata6* gene (exon 2) were deleted by adenoviral delivery of the Cre recombinase (AdCre). Fibroblasts from *Gata6fl* mouse hearts were infected with β-galactosidase expressing adenovirus (AdβGal) and used as control samples. Wild-type cardiac fibroblasts infected with AdCre served as additional controls. The genome-wide transcriptome was analyzed using bulk RNA-sequencing and differentially expressed (DE) genes between the groups (at least twofold up or downregulation in *Gata4/6* deletion compared to control, and *p* < 0.05) are shown in the heatmap (Fig. 5a). Compared to *Gata6fl-*AdβGal control samples, we found 3720 DE genes in fibroblasts with *Gata4* deletion, 667 DE genes upon *Gata6* deletion and 3154 DE genes in fibroblasts with *Gata4/6* double deletion. Visualization of upregulated genes upon *Gata4/6* single or double deletion in a Venn diagram showed that 60% of the upregulated genes in the *Gata4/6* double deleted cells were also upregulated in *Gata4* single deletion, while only 17% were upregulated in samples with *Gata6* single deletion (Fig. 5b). Because we did not observe any phenotype in mice with *Gata4* or *Gata6* single deletion in fibroblasts, we focused the analysis on the samples with *Gata4/6* double-deletion. In this group, we found 1408 upregulated genes which belonged to the GO (biological process) clusters blood vessel development, tissue morphogenesis and negative regulation of cell proliferation (Fig. 5a, Suppl. Figure 4a). Highly upregulated example genes in these clusters include angiogenic inhibitors (*Angpt4*, *Cnmd*, *Thbs1*), modulators of tissue morphogenesis (*Agt*, *Adamts12*), and cell receptors with anti-angiogenic effects (*Cd36*, *Flt1*, other example genes are listed in Fig. 5a). The expression levels of selected upregulated genes detected in the RNA-sequencing are shown in Fig. 5c. 1746 genes were downregulated in *Gata4/6* double-deleted fibroblasts compared to control cells and mainly belonged to the GO clusters virus response or interferon-beta response, for example *Cxcl10*, *Stat1*, and *Irf7* (Suppl. Figure 4b). Visualization of the gene clusters in a GO network shows the cluster inter-relation based on similarity (Fig. 5d), where the gene set annotated in the “blood vessel development” cluster (medium blue) shows high similarity (indicated by the number of connecting edges) with the gene sets of the clusters “positive regulation of cell migration” (left, purple) and “tissue morphogenesis” (right, lighter blue).

We subsequently focused on the upregulated genes annotated in blood vessel development and selected genes with a functional annotation in negative regulation of angiogenesis. Ranking of the 11 genes annotated in this functional class identified *Angpt4* and *Thbs1* as the two candidates meeting the following two selection criteria: highly different expression levels compared to control samples and high total expression levels in the *Gata4/6* double-deletion samples. The encoded proteins angiopoietin-4 (ANGPT4) and thrombospondin-1 (THBS1) are characterized as secreted factors, which enables anti-angiogenic effects without direct contact of the producing fibroblasts to the targeted endothelial cells, similar to the effect we observed in the co-culture system upon *Gata4/6* deletion in fibroblasts. Interestingly, the gene encoding the THBS1 receptor platelet glycoprotein 4 (*Cd36*) was also significantly upregulated in fibroblasts upon *Gata4/6* double-deletion, while single deletion of *Gata4* or *Gata6* caused lower changes in the expression level (upregulation *Gata4/6*-deletion 149%, *Gata4*-deletion 62%, *Gata6*-deletion 36% vs. control). Although CD36 mediates a variety of ligand and cell-type specific effects, a mechanistic link how elevated *Cd36* RNA expression level in fibroblasts might exert an anti-angiogenic effect on cardiac endothelial cells is so far undescribed. It is, however, well known that components of the extracellular matrix serve as ligands for the CD36 receptor [40].

### Deposition of the main extracellular matrix components is not affected by deletion of *Gata4* and *Gata6* in cardiac fibroblasts

**Fig. 6:**
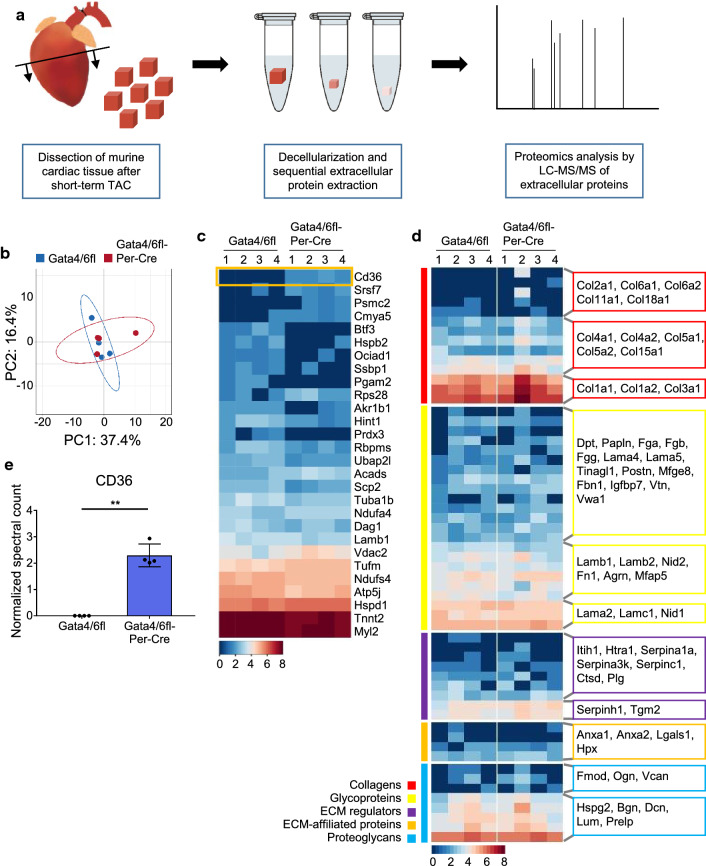
Proteomic profiling of the extracellular matrix (ECM) from whole hearts of *Gata4/6fl* and *Gata4/6fl-Per-Cre* mice shows significantly higher level of the anti-angiogenic glycoprotein CD36 in mice with fibroblast-specific downregulation of *Gata4* and *Gata6.* **a** Schematic overview of sample preparation for proteomic analysis of extracellular matrix components. **b** PCA plot of detected extracellular matrix proteins from hearts of *Gata4/6fl* and *Gata4/6fl-Per-Cre* mice. **c**, **d** Heat map of all significantly different protein levels (**c**) and of important ECM components shown in color-coded clusters (**d**) in the cardiac ECM of *Gata4/6fl* and *Gata4/6fl-Per-Cre* mice. Detected proteins are indicated by encoding gene names. Normalized spectral counts are shown as log2 protein levels, blue color coding represents lower protein abundance, whereas red represents high protein levels. The anti-angiogenic glycoprotein CD36 was detected as the most significantly upregulated ECM protein in *Gata4/6fl-Per-Cre* mice compared to controls (highlighted with an orange box in **c**). **e** Normalized counts of CD36 from the ECM proteomic analysis. Data are shown as mean ± SD. Unpaired *t* test with Welch’s correction was used for comparison of two groups in **e**. **p < 0.01

Following cardiac injury, activated cardiac fibroblasts are the main contributors to the deposition of extracellular matrix (ECM), which increases cardiac stiffness and thereby reduces the systolic and diastolic function of the heart. Although overall quantification of collagen deposition did not reveal quantitative differences in Sirius-red stained hearts of *Gata4/6fl-Per-Cre* and control mice after TAC, we sought to identify potential changes in the extracellular matrix composition that might affect cardiac function. Therefore, we isolated the extracellular matrix from whole hearts of *Gata4/6fl-Per-Cre* and *Gata4/6fl* control mice after short-term pressure overload and analyzed the proteins deposited in the extracellular matrix by mass spectrometry (Fig. 6a). Analysis of the primary components showed an overall similar composition of extracellular matrix proteins in mice with *Gata4/6* double deletion and controls (Fig. 6b). Further exploration of the main ECM proteins displayed no major difference in protein abundancies, especially for collagens, glycoproteins, and proteoglycans (Fig. 6d and Suppl. Table 3). We found only 28 proteins with significantly different levels in the ECM of *Gata4/6* double deletion and control hearts (Fig. 6c and Suppl. Table 4). Among them, the protein levels of the THBS1 receptor CD36 were the most significantly changed with no detectable deposition in control ECM and a mean of 2.3 normalized counts in the ECM of *Gata4/6fl-Per-Cre* hearts (Fig. 6c, e). CD36 is a transmembrane glycoprotein that can be secreted from cells, and that is expressed in various cell types, including (cardiac) fibroblasts, endothelial cells and macrophages [6, 14, 31, 46]. It functions to facilitate cellular long-chain fatty acid uptake, but also mediates anti-angiogenic signaling [10, 18]. Beside the marked change in CD36 abundance, the overall result of the ECM analysis did not indicate major changes in the ECM composition between *Gata4/6fl-Per-Cre* and control mice that would directly affect cardiac function. Therefore, we concluded that the reduced cardiac capillarization of *Gata4/6fl-Per-Cre* mice in response to pressure overload constitutes the key mechanism causing the deteriorated cardiac function compared to control animals.

### Increased secretion of angiogenic inhibitors ANGPT4 and THBS1 from *Gata4/6*-deleted cardiac fibroblasts mediates reduced angiogenesis

**Fig. 7:**
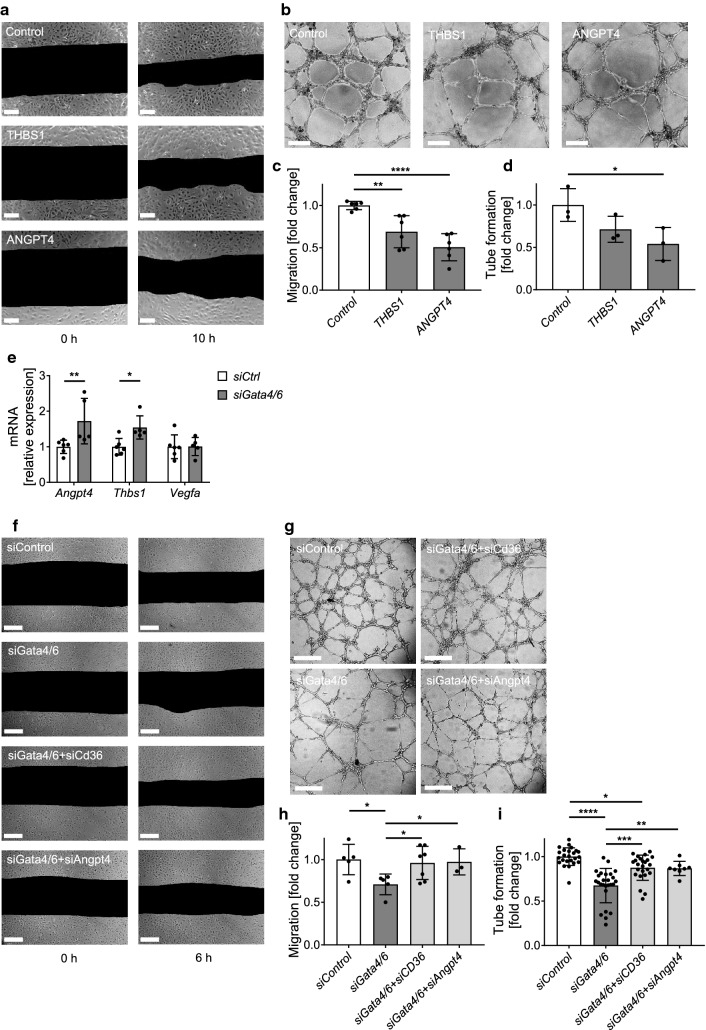
Anti-angiogenic effects of *Gata4* and *Gata6* downregulation in fibroblasts are mediated via CD36, thrombospondin-1 (THBS1) and angiopoietin-4 (ANGPT4). **a**, **c** Representative images of endothelial cell migration (**a**) and quantification (**c**) from scratch assays of HUVECs cultured in control medium (control) or in medium supplemented with recombinant THBS1 or ANGPT4 protein as indicated. Pictures were taken directly after scratch (0 h) and 10 h later. **b**, **d** Representative images showing the tube formation (**b**) and quantification of closed tubes (**d**) formed by HUVEC cultured on a 3D matrigel matrix. Images were taken after 8 h incubation in control medium (control) or medium supplemented with recombinant THBS1 or ANGPT4 protein. **e** Quantitative real-time-PCR of *Angpt4*, *Thbs1*, and *Vegfa* in fibroblasts after siRNA mediated downregulation of *Gata4* and *Gata6*. RNA expression levels are normalized to *Gapdh*. **f**, **h** Representative images of endothelial cell migration (**f**) and quantification (**h**) from scratch assay of HUVECs in co-culture with fibroblasts. *Gata4/6*, *Cd36* or *Angpt4* were downregulated in fibroblasts by siRNA as indicated. Pictures were taken directly after scratch (0 h) and 6 h later. **g**, **i** Representative images showing the tube formation (**g**) and quantification of closed tubes (**i**) of HUVEC/Fibroblast co-cultures on a 3D matrigel matrix. Images for quantification of tube formation were taken after 8 h of co-culture. Scale bar (**a**, **b**, **e,** and **f**) 200 µm. Data are shown as mean ± SD. One-way ANOVA with Tukey’s multiple-comparisons test (**c**, **d**, **h** and **i**) or student’s *t* test (**e**) was used to test for statistical significance. *p < 0.05, **p < 0.01, ***p < 0.001, ****p < 0.0001

To directly investigate the effects of secreted ANGPT4 or THBS1 on endothelial angiogenic function, we treated HUVECs with recombinant proteins. As expected, we observed reduced migration and tube formation in ANGPT4 or THBS1 treated cells compared to controls (Fig. 7a–d). To prove the relevance of the anti-angiogenic effect at endogenous protein concentrations, we again conducted co-culture experiments with siRNA transfected cardiac fibroblasts and HUVECs. As described before, *Gata4* and *Gata6* were simultaneously downregulated in cardiac fibroblasts, which caused significantly increased mRNA expression of *Angpt4* and *Thbs1* in cardiac fibroblasts, while expression levels of the angiogenic factor *Vegfa* that was previously described as target gene of *Gata4* in cardiomyocytes, was not significantly changed (Fig. 7e) [12]. Downregulation of *Gata4* and *Gata6* resulted in significantly reduced cell migration and tube formation of co-cultured HUVECs (Fig. 7f–i). Additional deletion of *Angpt4* in cardiac fibroblasts restored endothelial migration and tube formation almost to the level of control samples (Fig. 7f–i). Interestingly, siRNA mediated downregulation of *Cd36* in cardiac fibroblasts with *Gata4/6* double deletion also significantly improved the angiogenic response in co-cultured HUVECs (Fig. 7f–i). Taken together, these results point towards a novel signaling mechanism in the angiogenic response during pressure overload induced cardiac remodeling that involves not only intercellular crosstalk via secretion of angiogenesis-modulating ligands, but also extracellular deposition of signaling receptors that could modify the local ligand-availability.

## Discussion

In this study, we analyzed the role of the transcription factors GATA-4 and GATA-6 in cardiac fibroblasts during pressure overload induced cardiac remodeling. While single deletion of *Gata4* or *Gata6* in activated cardiac fibroblasts did not exert any phenotypic effect on cardiac function, we found a more decreased systolic function and signs of aggravated heart failure in mice with combined deletion of *Gata4* and *Gata6* in heart fibroblasts. Histological analysis revealed an impaired angiogenic response in the hearts of mice lacking *Gata4* and *Gata6* in cardiac fibroblasts. These results support the in vivo relevance of intercellular signaling mechanisms upon cardiac injury. Previous investigations on the role of GATA factors have found that single deletions of *Gata4* or *Gata6* can be partially or completely compensated by each other in several tissues and disease states [3, 38, 44, 47]. In line with these results, single deletion of *Gata4* in activated cardiac fibroblasts in our study had no detectable effect on the structural composition and function of the heart and single deletion of *Gata6* even caused an increased capillary density after pressure overload. While the increased angiogenic response in *Gata6* single deleted mice had no protective impact on the cardiac function, it might be a consequence of compensatory increase in GATA-4 signaling, again suggesting a functional redundancy of GATA-4 and GATA-6 in cardiac fibroblasts.

While especially GATA-4 has been extensively investigated in cardiomyocytes, where it acts as a central regulator during cardiac development, maladaptive hypertrophy and even provides regenerative properties, the fibroblast-specific functions of GATA-4 and GATA-6 are to our knowledge largely unknown [9, 25, 28, 30]. We found that among the GATA family of transcription factors, *Gata4* and *Gata6* show by far the highest RNA expression level in fibroblasts from adult mouse hearts and cardiac fibroblasts express at least equal, or even significantly higher levels of *Gata4* and *Gata6* compared to cardiomyocytes or cardiac endothelial cells. These findings confirm previous work, which had demonstrated that GATA-4 and GATA-6 are enriched in cardiac fibroblasts versus whole heart tissue, while they are not at all expressed in skin fibroblasts [8]. In fact, *Gata4* and *Gata6* together with *Tbx20* and *Hand2* were proposed to be part of a cardiogenic gene program in these cells. Although this suggested a heart specific role of this gene program in fibroblasts, its function in the myocardial stress response remained unknown.

Considering the major contribution of activated fibroblasts to the deposition of extracellular matrix components after pressure overload, we first investigated TAC induced fibrosis. While previous studies had shown that ablation of *Postn*-expressing cells prevented fibrosis and preserved the heart function after myocardial infarction or pharmacological stress, we did not observe any change in the total extent or the composition of cardiac connective tissue in hearts with double deletion of *Gata4/6* in activated fibroblasts compared to controls [19]. These results raised the question about additional, so far unknown functions of activated cardiac fibroblasts that could directly affect cardiac function during pathological overload. To enable continuous, heavy mechanical work, the heart strongly depends on oxidative metabolism and sustained energy supply that requires a dense network of blood vessels in close proximity to the working cardiomyocytes. While it is well established that hypertrophying cardiomyocytes stimulate capillary growth, at least in part via increased HIF-1α and GATA-4 dependent signaling, an intercellular crosstalk between fibroblasts and myocardial endothelial cells is less investigated [12]. In this study, we show that *Gata4/6* expression in cardiac fibroblasts promotes myocardial angiogenesis and thereby helps to maintain cardiac systolic function. Although we did not formally demonstrate a causal relation here, previous work had shown that reduction of cardiac angiogenesis impairs systolic heart function, while increased angiogenesis mediates the opposite [12, 17, 33, 36].

We identified two signaling pathways that mediate anti-angiogenic signaling from cardiac fibroblasts to endothelial cells upon reduction of fibroblast GATA-4/-6 levels: first, we found a dramatic increase in mRNA expression levels of *Angpt4* (angiopoietin-4) in cardiac fibroblasts upon *Gata4/6* deletion. While previous studies reported both, pro or anti-angiogenic functions of angiopoietin-4 depending on dose and study design, we show here that recombinant angiopoietin-4 clearly inhibits endothelial cell migration and tube formation and that siRNA mediated antagonism of the increased *Angpt4* RNA levels in cardiac fibroblasts restored angiogenic function in co-cultured HUVECs [22, 29]. Second, the *Thbs1* RNA levels as well as the expression of its receptor *Cd36* were upregulated in *Gata4/6*-depleted cardiac fibroblasts. While the glycoprotein thrombospondin-1 is well established as an anti-angiogenic secreted factor especially from activated platelets, the influence of increased *Cd36* expression levels in cardiac fibroblasts on neighboring endothelial cells remains somewhat unclear. Although we reproducibly found that counteractive downregulation of *Cd36* in fibroblasts with lack of *Gata4* and *Gata6* prevented the anti-angiogenic intercellular effect, further research will be needed to fully understand the underlying mechanisms. Because we detected strongly elevated CD36 deposition in the extracellular matrix of *Gata4/6fl-Per-Cre* mice, one might speculate that it serves as scaffold protein to increase local thrombospondin levels. A recent study from Vidal et al. found a similar anti-angiogenic gene set in single-nuclei sequencing from aged cardiac fibroblasts, which among other factors, also described the glycoprotein CD36 as one of the receptors mediating intercellular fibroblast endothelial crosstalk [45]. Besides the capability to mediate anti-angiogenic properties upon thrombospondin binding, one of the first identified functions of CD36 was the strong interaction with collagen [40]. Another possible function might, therefore, be that CD36 on the surface of fibroblasts is activated by collagen to induce a paracrine gene-program, which in turn inhibits capillary growth. Indeed, *Cd36* expressing fibroblasts counteract breast cancer cell growth in an organoid co-culture model by triggering the expression and release of unknown paracrine factors by fibroblasts [4]. However, as an in depth investigation of CD36 in cardiac fibroblasts exceeded the scope of this study, further work will be necessary to explore the mechanism of CD36 dependent effects in cardiac fibroblasts.

In this study, we focused on the transcription factors GATA-4 and GATA-6 to elucidate the role of the cardiac gene program in myocardial fibroblasts during pathological overload. Because compound deletion of *Gata4* and *Gata6* in cardiac fibroblasts reduced the adaptive angiogenic response during pressure overload and entailed cardiac dysfunction, we suggest that the cardiac gene-program in fibroblasts acts to maintain a myocardium specific interstitial milieu and high capillarization of the heart. The fibroblast-endothelial interface might therefore play a crucial role in the cardiac stress response and heart failure development and will require further research to enable targeted therapies in the future.

## Supplementary Information

Below is the link to the electronic supplementary material.

Supplementary file1 (DOCX 14 KB)

Supplementary file2 (DOCX 18 KB)

Supplementary file3 (XLSX 86 KB)

Supplementary file4 (XLSX 12 KB)

Supplementary file5 (PDF 668 KB)

## Data Availability

All data and material supporting the findings of this study are available from the corresponding author on request. RNA-seq data are available at Gene Expression Omnibus GSE155358.
