# Supplementary material for: Fibroblast GATA-4 and GATA-6 promote myocardial adaptation to pressure overload by enhancing cardiac angiogenesis
Source: Basic Res Cardiol. 2021 Apr 19;116(1):26. doi: 10.1007/s00395-021-00862-y (PMC8055639; doi:10.1007/s00395-021-00862-y)
Supplement: Supplementary file 1 — Supplementary file1 (DOCX 14 KB) [file 395_2021_862_MOESM1_ESM.docx]

| **Suppl. Table 1** Primer sequences used for qRT-PCR | | |  |
| --- | --- | --- | --- |
|  |  |  |  |
| **Gene** | **Species** | **forward primer (5'-3')** | **reverse primer (5'-3' on opposite strand)** |
| *Atp2a2* | Mouse | ACGTGCCTGGTGGAGAAGATGAAT | ATCTTGCTCATGGATGTCCGGCTT |
| *Col3a1* | Mouse | ATAAGCCCTGATGGTTCTCG | ATGCATGTTTCCCCAGTTTC |
| *Col11a1* | Mouse | AGAATTTCATCGTGGCCAAA | AGAGGCAGTCAGGAGCTTCA |
| *Gapdh* | Mouse | ACCCAGAAGACTGTGGATGG | CACATTGGGGGTAGGAACAC |
| *Gata1* | Mouse | ACTGTGGAGCAACGGCTACT | TCCGCCAGAGTGTTGTAGTG |
| *Gata2* | Mouse | TGCATGCAAGAGAAGTCACC | AGACTGGAGGAAGGGTGGAT |
| *Gata3* | Mouse | GTCATCCCTGAGCCACATCT | AGGGCTCTGCCTCTCTAACC |
| *Gata4* | Mouse | CTACCTGTGCAATGCCTGTG | GGTGGTGGTAGTCTGGCAGT |
| *Gata5* | Mouse | CGGCACCGGACACTATCTAT | CAGTATGGCAGTTGGAGCAG |
| *Gata6* | Mouse | AGTTTTCCGGCAGAGCAGTA | AGTCAAGGCCATCCACTGTC |
| *Myh6* | Mouse | ACTGTGGTGCCTCGTTCC | GCCTCTAGGCGTTCCTTCTC |
| *Myh7* | Mouse | AGGCAAGGCAAAGAAAGGCTCATC | GCGTGGAGCGCAAGTTTGTCATAA |
| *Nppa* | Mouse | ATTGACAGGATTGGAGCCCAGAGT | TGACACACCACAAGGGCTTAGGAT |
| *Nppb* | Mouse | CTCAAGCTGCTTTGGGCACAAGAT | AGCCAGGAGGTCTTCCTACAACAA |
| *Pdgfra* | Mouse | CTGCCAGACATTGACCCTGT | GAACCTGTCTCGATGGCACT |
| *Pecam1* | Mouse | TCCGGGAAGTACAAATGCACA | CGCCTTCTGTCACCTCCTTT |
| *Rcan1* | Mouse | GCTTGACTGAGGAGCGAGTC | CCACACAAGCAATCAGGGAGC |
| *Rpl7* | Mouse | TGGAACCATGGAGGCTGT | TCTCAGTGCGGTACATCTGC |
| *Tnnt2* | Mouse | CCTGGAGGCTGAGAAGTTCG | TTGGCCTTCCCACGAGTTTT |
| *Angpt4* | Rat | GACAGCAGCATCTCAGCAAG | GCGGATGCCATTGATCTTAT |
| *Gapdh* | Rat | ACCACCATGGAGAAGGCTGG | CTCAGTGTAGCCCAGGATGC |
| *Gata4* | Rat | TCAAACCAGAAAACGGAAGC | CTGCTGTGCCCATAGTGAGA |
| *Gata6* | Rat | CTTCTCCTCCCACACAGTCG | GTCGCACCGAGGATGTAACT |
| *Thbs1* | Rat | GCGGTAGACTAGGCCTGTTC | ATGCAAGAAGAGAGGCAGGG |
| *Vegfa* | Rat | CAAGATCCGCAGACGTGTAA | TCTAGTTCCCGAAACCCTGA |
