# Supplementary material for: Fibroblast GATA-4 and GATA-6 promote myocardial adaptation to pressure overload by enhancing cardiac angiogenesis
Source: Basic Res Cardiol. 2021 Apr 19;116(1):26. doi: 10.1007/s00395-021-00862-y (PMC8055639; doi:10.1007/s00395-021-00862-y)
Supplement: Supplementary file 2 — Supplementary file2 (DOCX 18 KB) [file 395_2021_862_MOESM2_ESM.docx]

| **Suppl. Table 2** Echocardiography parameters | |  |  |  |
| --- | --- | --- | --- | --- |
| **a** |  |  |  |  |
|  | **Gata4fl** | **Gata4fl-** | **Gata4fl** | **Gata4fl-** |
|  |  | **Per-Cre** |  | **Per-Cre** |
|  | **Sham** | **Sham** | **Short TAC** | **Short TAC** |
|  | n=4 | n=3 | n=13 | n=8 |
| **Heart Rate [BPM)** | 515.25±42.91 | 521.00±36.01 | 517.08±25.63 | 521.63±42.57 |
| **Systolic Diameter [mm]** | 2.07±0.09 | 2.41±0.19 | 2.74±0.25 | 2.33±0.43 |
| **Diastolic Diameter [mm]** | 3.49±0.54 | 3.31±0.15 | 3.90±0.33 | 3.66±0.27 |
| **Systolic Area [mm²]** | 3.82±0.31 | 4.53±0.83 | 5.99±1.10 | 4.84±1.63 |
| **Diastolic Area [mm²]** | 9.36±2.02 | 8.53±1.07 | 11.71±1.64 | 10.67±2.61 |
| **Fractional Area Change [%]** | 58.12±7.04 | 45.72±6.41 | 48.82±7.18 | 54.98±8.84 |
| **Ejection Fraction [%]** | 70.75±6.46 | 59.62±7.27 | 63.07±8.25 | 68.54±9.65 |
|  |  |  |  |  |
| **b** |  |  |  |  |
|  | **Gata6fl** | **Gata6fl-** | **Gata6fl** | **Gata6fl-** |
|  |  | **Per-Cre** |  | **Per-Cre** |
|  | **Sham** | **Sham** | **Short TAC** | **Short TAC** |
|  | n=8 | n=4 | n=9 | n=11 |
| **Heart Rate [BPM)** | 448.13±51.08 | 452.75±38.25 | 519.44±56.12 | 516.64±42.39 |
| **Systolic Diameter [mm]** | 2.28±0.43 | 2.51±0.31 | 3.58±0.49 | 3.64±0.51 |
| **Diastolic Diameter [mm]** | 3.28±0.29 | 3.43±0.23 | 4.19±0.44 | 4.39±0.50 |
| **Systolic Area [mm²]** | 4.42±1.47 | 4.84±0.86 | 9.81±2.85 | 10.01±3.27 |
| **Diastolic Area [mm²]** | 8.30±1.00 | 9.27±0.46 | 13.59±3.03 | 14.05±3.44 |
| **Fractional Area Change [%]** | 47.89±12.56 | 47.46±10.61 | 28.59±5.86 | 29.91±6.17 |
| **Ejection Fraction [%]** | 60.28±14.21 | 62.45±10.35 | 40.00±7.52 | 40.36±8.85 |
|  |  |  |  |  |
| **c** |  |  |  |  |
|  | **Gata4/6wt** | **Gata4/6wt-** | **Gata4/6wt** | **Gata4/6wt-** |
|  |  | **Per-Cre** |  | **Per-Cre** |
|  | **Sham** | **Sham** | **Short TAC** | **Short TAC** |
|  | n=4 | n=4 | n=6 | n=6 |
| **Heart Rate [BPM)** | 469.50±73.52 | 486.75±28.37 | 526.67±56.43 | 522.67±73.86 |
| **Systolic Diameter [mm]** | 3.42±0.23 | 2.96±0.46 | 3.99±0.53 | 4.45±0.82 |
| **Diastolic Diameter [mm]** | 4.27±0.42 | 3.73±0.29 | 4.62±0.59 | 4.90±0.78 |
| **Systolic Area [mm²]** | 9.64±1.23 | 7.58±2.23 | 12.30±3.22 | 15.73±4.90 |
| **Diastolic Area [mm²]** | 14.06±2.27 | 11.23±1.59 | 16.08±4.27 | 19.33±5.28 |
| **Fractional Area Change [%]** | 31.00±5.12 | 33.00±14.49 | 23.48±2.49 | 19.47±5.76 |
| **Ejection Fraction [%]** | 40.52±7.02 | 43.02±16.84 | 32.16±4.79 | 28.32±7.11 |
|  |  |  |  |  |
|  |  |  |  |  |
| **d** |  |  |  |  |
|  | **Gata4/6fl** | **Gata4/6fl-** | **Gata4/6fl** | **Gata4/6fl-** |
|  |  | **Per-Cre** |  | **Per-Cre** |
|  | **Short Sham** | **Short Sham** | **Short TAC** | **Short TAC** |
|  | n=7 | n=8 | n=18 | n=14 |
| **Heart Rate [BPM)** | 468.10±58.77 | 471.47±34.55 | 483.67±51.42 | 474.48±57.03 |
| **Systolic Diameter [mm]** | 3.49±0.47 | 3.49±0.43 | 4.66±0.61 | 4.92±0.56 |
| **Diastolic Diameter [mm]** | 4.93±0.28 | 4.81±0.37 | 5.71±0.50 | 5.67±0.51 |
| **Systolic Area [mm²]** | 9.73±2.54 | 9.69±2.28 | 17.30±4.27 | 19.26±4.31 |
| **Diastolic Area [mm²]** | 19.12±2.19 | 18.28±2.77 | 25.83±4.41 | 25.48±4.44 |
| **Fractional Area Change [%]** | 54.28±10.16 | 55.41±7.86 | 40.57±13.00 | 29.32±9.19 * |
| **Ejection Fraction [%]** | 63.27±11.35 | 61.59±8.24 | 45.05±12.99 | 34.57±9.22 * |
| *p<0.05 vs Gata4/6fl Short TAC |  |  |  |  |
| **e** |  |  |  |  |
|  | **Gata4/6fl** | **Gata4/6fl-** | **Gata4/6fl** | **Gata4/6fl-** |
|  |  | **Per-Cre** |  | **Per-Cre** |
|  | **Long Sham** | **Long Sham** | **Long TAC** | **Long TAC** |
|  | n=9 | n=7 | n=13 | n=6 |
| **Heart Rate [BPM)** | 541.1±44.5 | 578.7±23.7 | 518.32±47.54 | 539.25±45.97 |
| **Systolic Diameter [mm]** | 2.5±0.5 | 2.5±0.3 | 4.68±0.45 | 4.62±0.62 |
| **Diastolic Diameter [mm]** | 3.9±0.4 | 3.7±0.3 | 5.40±0.42 | 5.04±0.70 |
| **Systolic Area [mm²]** | 5.0±2.2 | 5.0±1.3 | 17.35±3.41 | 16.98±4.47 |
| **Diastolic Area [mm²]** | 12.2±2.8 | 11.0±1.8 | 23.00±3.56 | 20.27±5.38 |
| **Fractional Area Change [%]** | 59.3±14.4 | 55.1±5.4 | 26.26±8.17 | 17.58±2.93 * |
| **Ejection Fraction [%]** | 73.0±13.5 | 70.2±6.4 | 34.59±8.13 | 22.93±5.21 ** |
| *p<0.05 vs Gata4/6fl Long TAC **p<0.01 vs Gata4/6fl Long TAC | | |  |  |
|  |  |  |  |  |
