# Supplementary material for: Fibroblast GATA-4 and GATA-6 promote myocardial adaptation to pressure overload by enhancing cardiac angiogenesis
Source: Basic Res Cardiol. 2021 Apr 19;116(1):26. doi: 10.1007/s00395-021-00862-y (PMC8055639; doi:10.1007/s00395-021-00862-y)
Supplement: Supplementary file 5 — Supplementary file5 (PDF 668 KB) [file 395_2021_862_MOESM5_ESM.pdf]

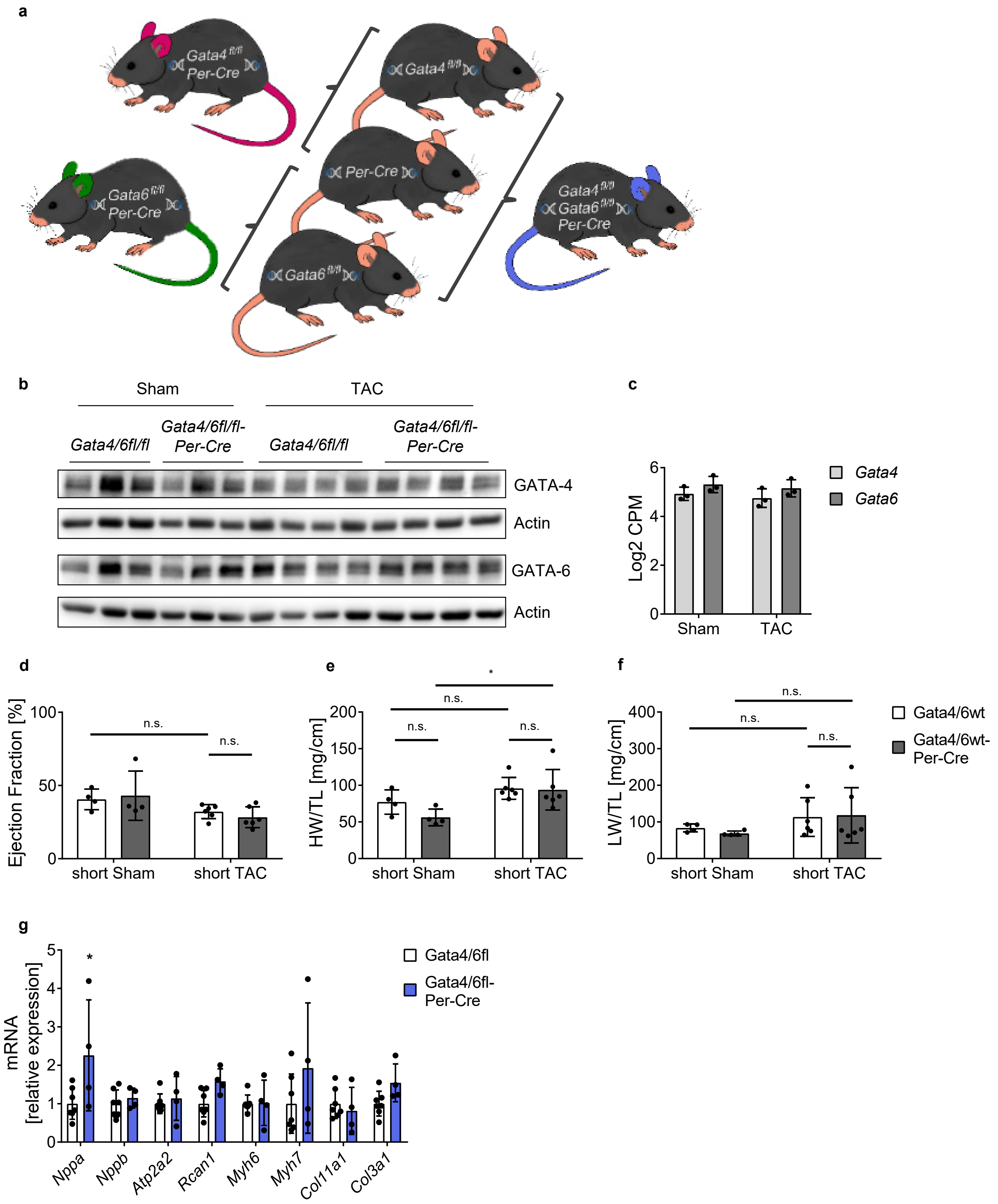

Suppl. Fig. 1 a Schematic illustration showing the interbreeding of *Gata4*$^{fl/fl}$ and *Gata6*$^{fl/fl}$ with *Tg(Postn-Cre)* (short: *Per-Cre*) mice to obtain mice with specific deletion of *Gata4* (pink), *Gata6* (green) and *Gata4/6* (blue) in activated cardiac fibroblasts. **b** Total GATA-4 and GATA-6 protein level in whole heart tissue from *Gata4/6-Per-Cre* and control mice. **c** Quantification of *Gata4* and *Gata6* RNA expression levels in isolated cardiac fibroblasts from RNA-sequencing (displayed as counts per million reads (CPM)) shows no significant change after pressure overload compared to sham. **d-f** Quantification of the ejection fraction (**d**), HW/TL (**e**) and LW/TL ratio (**f**) in *Gata4/6wt-Per-Cre* and *Gata4/6wt* littermate control mice shows no significant differences in both groups after short-term pressure overload. **g** Relative mRNA expression levels of embryonic cardiac genes and collagen genes in *Gata4/6fl-Per-Cre* and control mice after long-term pressure overload. RNA level are normalized to Gapdh. Data are shown as mean ± SD. 2-way ANOVA with Sidak's multiple comparisons test (**c-f**) or student's t-test (**g**) was used to test for statistical significance. n.s. indicates no statistical significance between groups, *p<0.05.

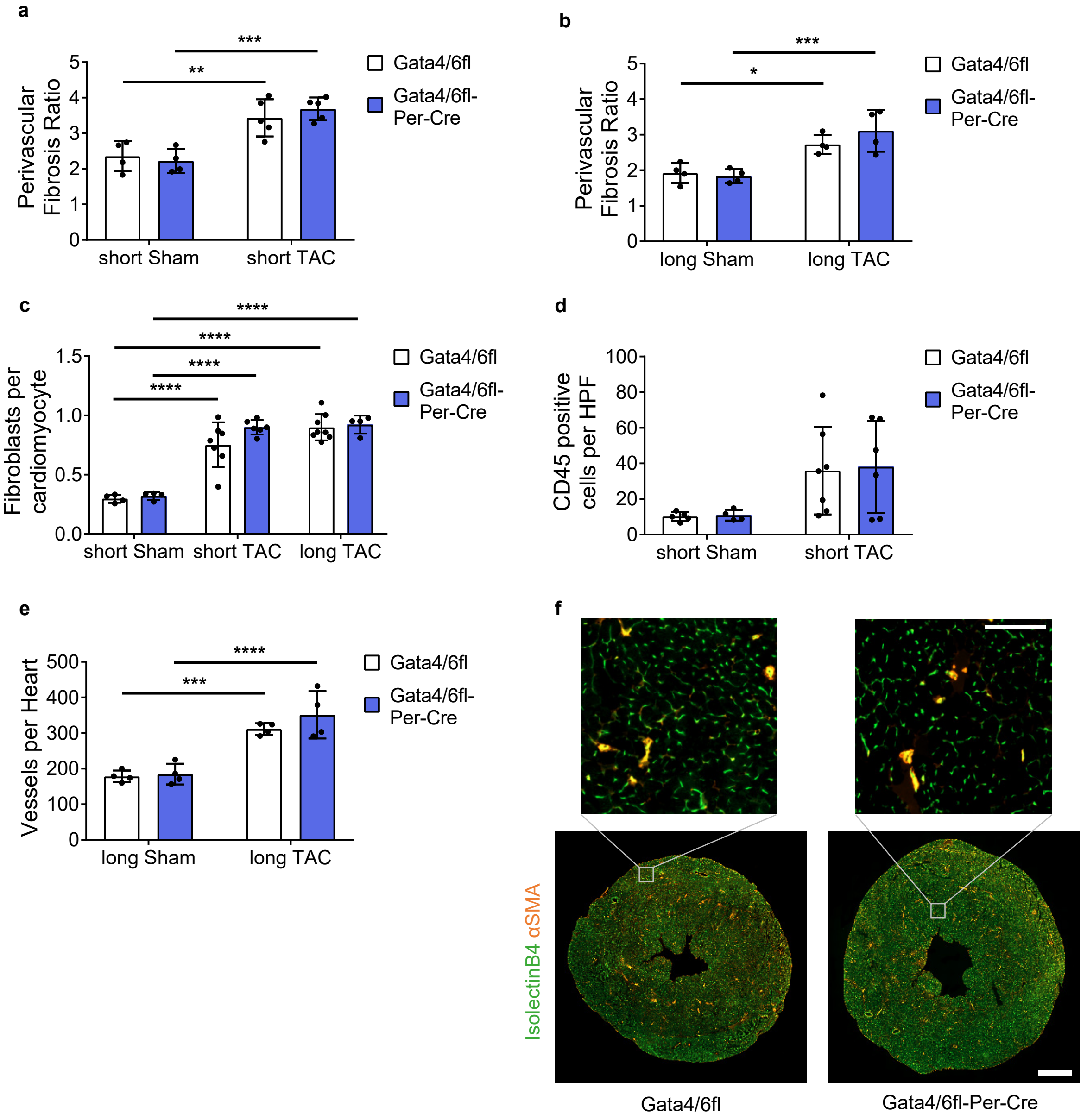

Suppl. Fig. 2 a, b Perivascular fibrosis ratio in heart sections of *Gata4/6fl-Per-Cre* and control mice after short-term (**a**) and long-term (**b**) pressure overload. **c** Number of cardiac fibroblasts per cardiomyocyte in heart sections of *Gata4/6fl-Per-Cre* and control mice quantified from immunofluorescence staining with WGA, PDGFRα and DAPI. **d** Number of CD45 positive cells per high-power field (HPF) in heart sections of *Gata4/6fl-Per-Cre* and control mice. At least 3 images were quantified per heart. **e, f** Number of smooth muscle alpha-actin (αSMA) positive vessels per heart cross section (**e**) and representative images of αSMA/IB4 staining after long TAC (**f**), scale bar 1 mm for whole heart images and 100 µm for zoomed areas. Data are shown as mean ± SD. 2-way ANOVA with Sidak's multiple comparisons test was used to test for statistical significance. *$p<0.05$, **$p<0.01$, ***$p<0.001$, ****$p<0.0001$.

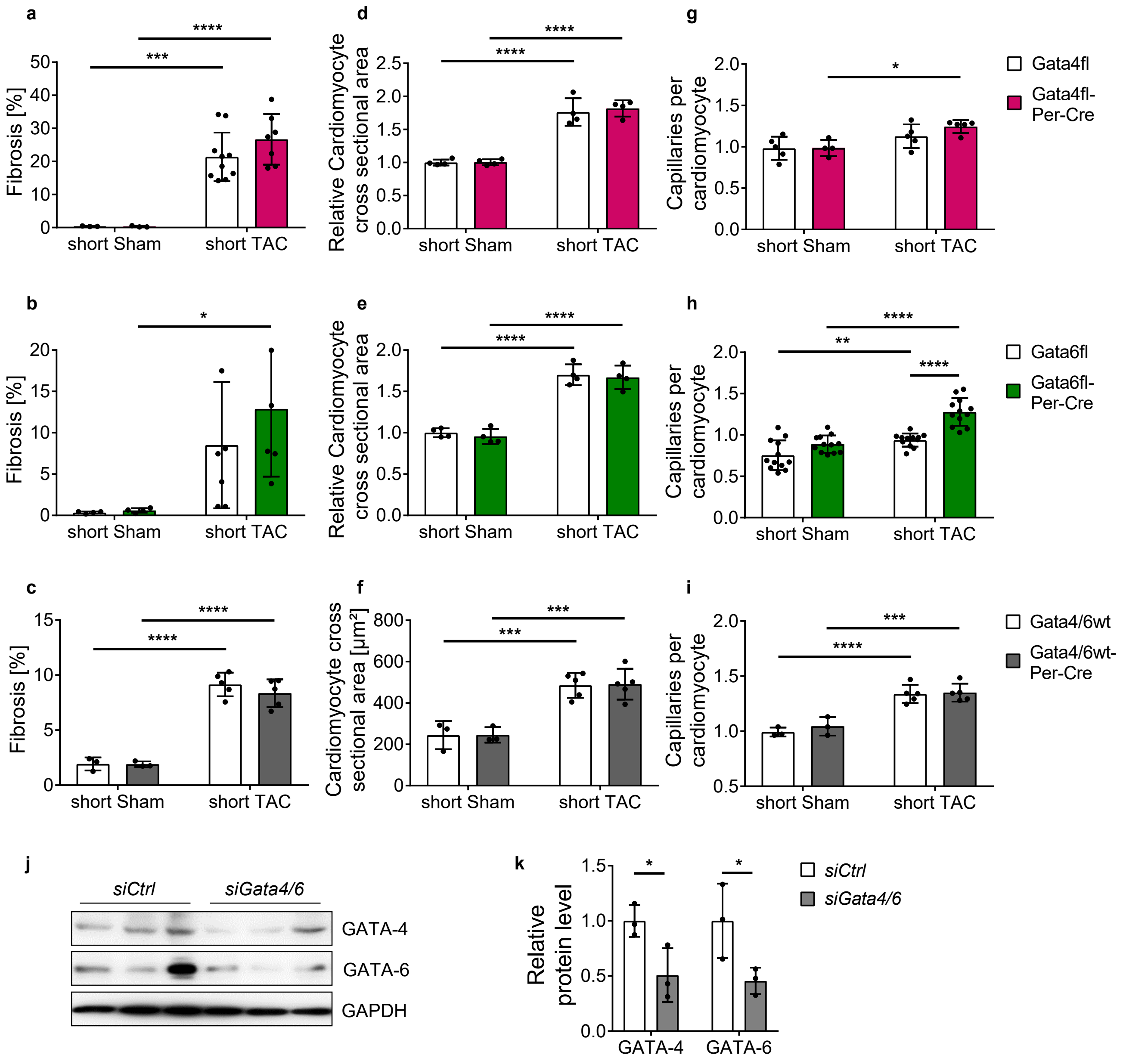

Suppl. Fig. 3 a-c Quantification of cardiac fibrosis from transverse mouse heart sections stained with Sirius red after short term pressure overload or sham in *Gata4fl-Per-Cre* (**a**), *Gata6fl-Per-Cre* (**b**) and *Gata4/6wt-Per-Cre* (**c**) mice. **d-f** Quantification of the cardiomyocyte cross sectional area after short term pressure overload or sham in *Gata4fl-Per-Cre* (**d**), *Gata6fl-Per-Cre* (**e**) and *Gata4/6wt-Per-Cre* mice (**f**). **g-i** Quantification of the capillary density displayed as capillary per cardiomyocyte ratio after short term pressure overload or sham in *Gata4fl-Per-Cre* (**g**), *Gata6fl-Per-Cre* (**h**) and *Gata4/6wt-Per-Cre* (**i**) mice. **j**, **k** GATA-4 and GATA-6 protein levels in rat cardiac fibroblasts after siRNA mediated downregulation (**j**) and quantification (**k**) showing efficient deletion of GATA-4 and GATA-6. Protein levels were normalized to GAPDH for quantification. Data are shown as mean ± SD. 2-way ANOVA with Sidak's multiple comparisons test (a-i) or student's t-test (**k**) was used to test for statistical significance. *p<0.05, **p<0.01, ***p<0.001, ****p<0.0001.

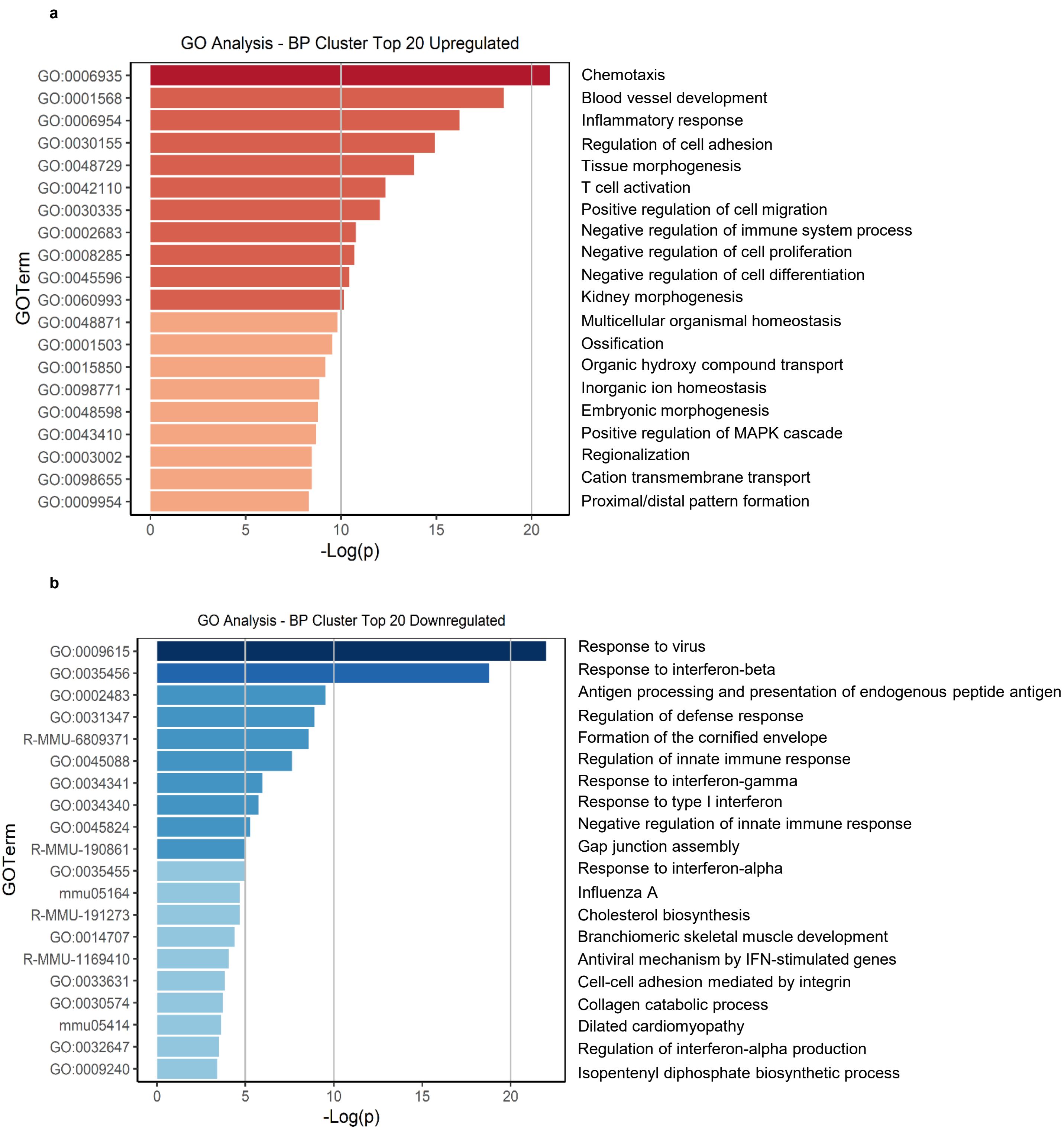

Suppl. Fig. 4 TOP 20 gene ontology cluster showing the highest enriched genes upon *Gata4/6* deletion in cardiac fibroblasts. **a** GO BP cluster of upregulated genes upon *Gata4/6* deletion. **b** GO BP cluster of downregulated genes upon *Gata4/6* deletion. Analysis was performed with metascape.
